# (1S*,2R*,4aS*,6aS*,6bR*,10S*,12aR*,14aS*)-10-Hydr­oxy-1,2,6a,6b,9,9,12a-hepta­methyl­perhydro­picene-4a,14a-carbolactone

**DOI:** 10.1107/S1600536809008253

**Published:** 2009-03-14

**Authors:** Dan-Wei Ou-yang, Jian-Ping Gao, Qing-Shan Li, Jian-Ping Guo

**Affiliations:** a Institute of Pharmaceutical Science, Shanxi Medical University, 56 South Xinjian Road, Taiyuan 030001, People’s Republic of China; b Institute of Chemistry and Engineering, Shanxi University, 96 Wucheng Road, Taiyuan 030006, People’s Republic of China

## Abstract

The title compound, C_30_H_48_O_3_, was extracted from the plant *Dracocephalum rupestre* Hance. The mol­ecule contains five fused cyclo­hexane rings and one five-membered lactone ring. Inter­molecular O—H⋯O hydrogen bonds between the hydroxyl and carbonyl groups link the mol­ecules into chains along [010]. The absolute structure has not been determined.

## Related literature

For related literature concerning the title compound and the plant *Dracocephalum rupestre *Hance, see: Jiangsu College of New Medicine (1977 ▶); Katai *et al.* (1983 ▶).
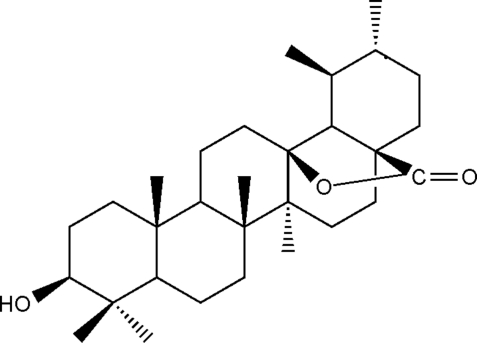


## Experimental

### 

#### Crystal data

C_30_H_48_O_3_
*M* *_r_* = 456.68
Monoclinic, 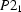

*a* = 8.156 (3) Å
*b* = 12.005 (5) Å
*c* = 13.475 (5) Å
β = 90.520 (7)°
*V* = 1319.3 (9) Å^3^
*Z* = 2
Mo *K*α radiation
μ = 0.07 mm^−1^
*T* = 293 K
0.60 × 0.50 × 0.30 mm

#### Data collection

Bruker SMART CCD diffractometer
Absorption correction: multi-scan (*SADABS*; Sheldrick, 1996 ▶) *T*_min_ = 0.958, *T*_max_ = 0.979
5150 measured reflections
2428 independent reflections
2052 reflections with *I* > 2σ(*I*)
*R*_int_ = 0.032

#### Refinement

*R*[*F* ^2^ > 2σ(*F* ^2^)] = 0.052
*wR*(*F* ^2^) = 0.142
*S* = 1.03
2428 reflections
299 parameters
1 restraint
H-atom parameters constrained
Δρ_max_ = 0.24 e Å^−3^
Δρ_min_ = −0.25 e Å^−3^

### 

Data collection: *SMART* (Bruker, 2000 ▶); cell refinement: *SAINT* (Bruker, 2000 ▶); data reduction: *SAINT*; program(s) used to solve structure: *SHELXS97* (Sheldrick, 2008 ▶); program(s) used to refine structure: *SHELXL97* (Sheldrick, 2008 ▶); molecular graphics: *SHELXTL* (Sheldrick, 2008 ▶); software used to prepare material for publication: *SHELXTL*.

## Supplementary Material

Crystal structure: contains datablocks I, global. DOI: 10.1107/S1600536809008253/bi2335sup1.cif

Structure factors: contains datablocks I. DOI: 10.1107/S1600536809008253/bi2335Isup2.hkl

Additional supplementary materials:  crystallographic information; 3D view; checkCIF report

## Figures and Tables

**Table 1:** Hydrogen-bond geometry (Å, °)

*D*—H⋯*A*	*D*—H	H⋯*A*	*D*⋯*A*	*D*—H⋯*A*
O3—H3*A*⋯O2^i^	0.82	2.24	3.059 (4)	176

Symmetry code: (i) 
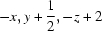
.

**Fig. 1.**
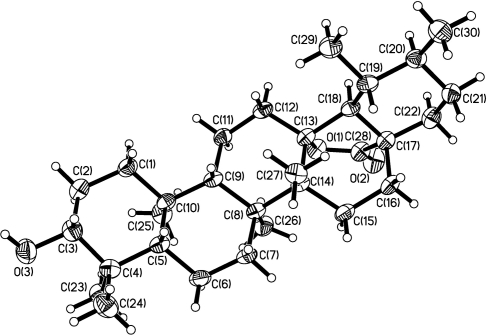
Molecular structure showing displacement ellipsoids at 50% probabilty for non-H atoms.

## supplementary crystallographic information

### Comment

The title compound is extracted from the plant *Dracocephalum rupestre Hance* (Jiangsu College of New Medicine, 1977) with ethanol. The compound (Katai *et al.*, 1983) was successfully crystallized from methanol. There are five six-membered rings and one five-membered ring in the molecule. The six-membered rings are composed of *sp*^3^-hybridised C and the five-membered ring is a lactone in which C28 is *sp*^2^ hybridised. The bond distances between C28 and O are 1.349 (3) [O1—C28] and 1.218 (4) Å [O2—C28]. The O2—C28 bond length of 1.216 (5) Å is a typical C=O double bond.

### Experimental

The dry aerial part of the plant (5.3 kg) was extracted with 95% ethanol 3 times under reflux. The ethanol extract was diluted with a large amount of water,and then extracted with petroleum ether, chloroform, EtOAc and n-butanol. The chloroform fraction (70 g) was subjected to Si gel column (1.5 kg,200–300 mesh) chromatography eluting with a gradient (petroleum ether-EtOAc, 99:1, 98:2, 97:3, 95:5, 9:1, 8:2,7:3, 1:1, *v*/*v*) to obtain 8 fractions (F1—F8). Fraction F3 (19.4 g) was separated by Si gel column (500 g, 200–300 mesh) chromatography eluting with a gradient (chloroform-methanol, 99:1, 98:2, 97:3, 95:5, 9:1,8:2,7:3, 1:1, *v*/*v*) to yield four portions. Subfraction 1 was subsequently subjected to Si gel column chromatography eluting with chloroform-methanol (10:1), and recrystallized from methanol, to obtain the title compound (25 mg).

### Refinement

H atoms were placed geometrically and allowed to ride with *U*_iso_(H) = 1.2 or 1.5*U*_eq_(C/O). In the absence of significant anomalous scattering, Friedel pairs were merged as equivalent data, and the absolute structure has not been determined.

### Crystal data

**Table:**

C_30_H_48_O_3_	*F*(000) = 504
*M**_r_* = 456.68	*D*_x_ = 1.150 Mg m^−^^3^
Monoclinic, *P*2_1_	Melting point: 519 K
Hall symbol: P 2yb	Mo *K*α radiation, λ = 0.71073 Å
*a* = 8.156 (3) Å	Cell parameters from 2717 reflections
*b* = 12.005 (5) Å	θ = 2.3–26.2°
*c* = 13.475 (5) Å	µ = 0.07 mm^−^^1^
β = 90.520 (7)°	*T* = 293 K
*V* = 1319.3 (9) Å^3^	Block, colourless
*Z* = 2	0.60 × 0.50 × 0.30 mm

### Data collection

**Table:**

Bruker SMART CCD diffractometer	2428 independent reflections
Radiation source: fine-focus sealed tube	2052 reflections with *I* > 2σ(*I*)
graphite	*R*_int_ = 0.032
ω scans	θ_max_ = 25.0°, θ_min_ = 1.5°
Absorption correction: multi-scan (*SADABS*; Sheldrick, 1996)	*h* = −9→7
*T*_min_ = 0.958, *T*_max_ = 0.979	*k* = −14→14
5150 measured reflections	*l* = −13→16

### Refinement

**Table:**

Refinement on *F*^2^	Primary atom site location: structure-invariant direct methods
Least-squares matrix: full	Secondary atom site location: difference Fourier map
*R*[*F*^2^ > 2σ(*F*^2^)] = 0.052	Hydrogen site location: inferred from neighbouring sites
*wR*(*F*^2^) = 0.142	H-atom parameters constrained
*S* = 1.03	*w* = 1/[σ^2^(*F*_o_^2^) + (0.0816*P*)^2^ + 0.1773*P*] where *P* = (*F*_o_^2^ + 2*F*_c_^2^)/3
2428 reflections	(Δ/σ)_max_ < 0.001
299 parameters	Δρ_max_ = 0.24 e Å^−^^3^
1 restraint	Δρ_min_ = −0.24 e Å^−^^3^

### Special details

**Table:**

Geometry. All e.s.d.'s (except the e.s.d. in the dihedral angle between two l.s. planes) are estimated using the full covariance matrix. The cell e.s.d.'s are taken into account individually in the estimation of e.s.d.'s in distances, angles and torsion angles; correlations between e.s.d.'s in cell parameters are only used when they are defined by crystal symmetry. An approximate (isotropic) treatment of cell e.s.d.'s is used for estimating e.s.d.'s involving l.s. planes.
Refinement. Refinement of *F*^2^ against ALL reflections. The weighted *R*-factor *wR* and goodness of fit *S* are based on *F*^2^, conventional *R*-factors *R* are based on *F*, with *F* set to zero for negative *F*^2^. The threshold expression of *F*^2^ > σ(*F*^2^) is used only for calculating *R*-factors(gt) *etc*. and is not relevant to the choice of reflections for refinement. *R*-factors based on *F*^2^ are statistically about twice as large as those based on *F*, and *R*- factors based on ALL data will be even larger.

### Fractional atomic coordinates and isotropic or equivalent isotropic displacement parameters (Å^2^)

**Table:**

	*x*	*y*	*z*	*U*_iso_*/*U*_eq_	
O1	−0.1317 (3)	0.74752 (19)	0.69165 (16)	0.0521 (6)	
O2	−0.1163 (4)	0.6395 (2)	0.5579 (2)	0.0776 (9)	
O3	0.2976 (4)	0.9857 (3)	1.2991 (2)	0.0794 (9)	
H3A	0.2459	1.0276	1.3353	0.119*	
C1	−0.0162 (4)	0.9125 (4)	1.1007 (3)	0.0566 (9)	
H1A	−0.0476	0.9870	1.0801	0.068*	
H1B	−0.1154	0.8681	1.1051	0.068*	
C2	0.0658 (5)	0.9185 (4)	1.2036 (3)	0.0654 (10)	
H2A	−0.0095	0.9529	1.2497	0.078*	
H2B	0.0879	0.8435	1.2268	0.078*	
C3	0.2236 (5)	0.9837 (3)	1.2033 (3)	0.0592 (9)	
H3B	0.1969	1.0606	1.1852	0.071*	
C4	0.3498 (4)	0.9406 (3)	1.1284 (3)	0.0543 (8)	
C5	0.2606 (4)	0.9273 (3)	1.0255 (2)	0.0464 (8)	
H5A	0.2293	1.0034	1.0072	0.056*	
C6	0.3736 (4)	0.8899 (4)	0.9416 (3)	0.0590 (9)	
H6A	0.4776	0.9287	0.9472	0.071*	
H6B	0.3946	0.8107	0.9475	0.071*	
C7	0.2955 (4)	0.9145 (4)	0.8405 (3)	0.0583 (9)	
H7A	0.3690	0.8888	0.7891	0.070*	
H7B	0.2838	0.9945	0.8332	0.070*	
C8	0.1261 (4)	0.8596 (3)	0.8242 (2)	0.0466 (8)	
C9	0.0174 (3)	0.8830 (3)	0.9165 (2)	0.0432 (7)	
H9A	−0.0032	0.9634	0.9147	0.052*	
C10	0.0967 (4)	0.8611 (3)	1.0212 (2)	0.0457 (7)	
C11	−0.1523 (4)	0.8290 (3)	0.9008 (2)	0.0510 (8)	
H11A	−0.1393	0.7492	0.8930	0.061*	
H11B	−0.2190	0.8421	0.9589	0.061*	
C12	−0.2392 (4)	0.8762 (3)	0.8096 (2)	0.0527 (8)	
H12A	−0.3415	0.8363	0.7998	0.063*	
H12B	−0.2660	0.9536	0.8222	0.063*	
C13	−0.1418 (4)	0.8697 (3)	0.7150 (2)	0.0439 (7)	
C14	0.0382 (4)	0.9125 (3)	0.7273 (2)	0.0430 (7)	
C15	0.1341 (4)	0.8817 (4)	0.6322 (2)	0.0550 (9)	
H15A	0.2303	0.9289	0.6284	0.066*	
H15B	0.1718	0.8053	0.6382	0.066*	
C16	0.0360 (4)	0.8931 (3)	0.5351 (2)	0.0532 (9)	
H16A	0.0952	0.8559	0.4825	0.064*	
H16B	0.0282	0.9714	0.5179	0.064*	
C17	−0.1378 (4)	0.8442 (3)	0.5403 (2)	0.0477 (8)	
C18	−0.2370 (4)	0.9073 (3)	0.6187 (2)	0.0445 (7)	
H18A	−0.3417	0.8675	0.6216	0.053*	
C19	−0.2835 (4)	1.0265 (3)	0.5865 (2)	0.0496 (8)	
H19A	−0.1825	1.0694	0.5774	0.059*	
C20	−0.3750 (4)	1.0192 (3)	0.4849 (2)	0.0530 (8)	
H20A	−0.4761	0.9767	0.4949	0.064*	
C21	−0.2730 (5)	0.9578 (4)	0.4078 (3)	0.0577 (9)	
H21A	−0.1745	1.0005	0.3949	0.069*	
H21B	−0.3353	0.9533	0.3462	0.069*	
C22	−0.2241 (5)	0.8403 (3)	0.4399 (3)	0.0592 (9)	
H22A	−0.3210	0.7939	0.4441	0.071*	
H22B	−0.1516	0.8080	0.3910	0.071*	
C23	0.4318 (5)	0.8333 (4)	1.1653 (3)	0.0712 (11)	
H23A	0.4847	0.8471	1.2281	0.107*	
H23B	0.5118	0.8094	1.1181	0.107*	
H23C	0.3504	0.7763	1.1730	0.107*	
C24	0.4838 (5)	1.0301 (4)	1.1204 (3)	0.0748 (12)	
H24A	0.5386	1.0381	1.1833	0.112*	
H24B	0.4348	1.0998	1.1018	0.112*	
H24C	0.5616	1.0083	1.0710	0.112*	
C25	0.1145 (5)	0.7347 (3)	1.0452 (3)	0.0612 (9)	
H25A	0.1114	0.7239	1.1157	0.092*	
H25B	0.2171	0.7080	1.0202	0.092*	
H25C	0.0261	0.6944	1.0144	0.092*	
C26	0.1588 (5)	0.7321 (3)	0.8123 (3)	0.0622 (10)	
H26A	0.2380	0.7085	0.8611	0.093*	
H26B	0.2002	0.7176	0.7471	0.093*	
H26C	0.0584	0.6918	0.8214	0.093*	
C27	0.0327 (4)	1.0412 (3)	0.7341 (3)	0.0542 (8)	
H27A	−0.0192	1.0708	0.6755	0.081*	
H27B	0.1423	1.0698	0.7395	0.081*	
H27C	−0.0286	1.0629	0.7914	0.081*	
C28	−0.1268 (4)	0.7329 (3)	0.5923 (3)	0.0546 (8)	
C29	−0.3888 (5)	1.0861 (4)	0.6629 (3)	0.0715 (11)	
H29A	−0.3304	1.0900	0.7250	0.107*	
H29B	−0.4892	1.0456	0.6718	0.107*	
H29C	−0.4132	1.1600	0.6401	0.107*	
C30	−0.4225 (5)	1.1339 (4)	0.4454 (3)	0.0727 (11)	
H30A	−0.4788	1.1258	0.3830	0.109*	
H30B	−0.3254	1.1778	0.4363	0.109*	
H30C	−0.4930	1.1702	0.4919	0.109*	

### Atomic displacement parameters (Å^2^)

**Table:**

	*U*^11^	*U*^22^	*U*^33^	*U*^12^	*U*^13^	*U*^23^
O1	0.0634 (14)	0.0347 (12)	0.0582 (13)	−0.0028 (11)	0.0101 (10)	0.0014 (10)
O2	0.115 (2)	0.0379 (14)	0.0798 (18)	0.0061 (15)	0.0098 (16)	−0.0079 (13)
O3	0.088 (2)	0.084 (2)	0.0662 (16)	0.0096 (17)	−0.0086 (15)	−0.0169 (15)
C1	0.0500 (19)	0.064 (2)	0.056 (2)	−0.0025 (17)	0.0118 (15)	−0.0011 (17)
C2	0.067 (2)	0.072 (3)	0.057 (2)	0.002 (2)	0.0129 (18)	−0.0041 (18)
C3	0.066 (2)	0.051 (2)	0.060 (2)	0.0044 (18)	−0.0036 (17)	−0.0040 (16)
C4	0.0513 (19)	0.049 (2)	0.063 (2)	0.0008 (16)	−0.0003 (16)	0.0036 (16)
C5	0.0414 (16)	0.0422 (18)	0.0556 (19)	0.0017 (14)	0.0075 (14)	0.0048 (14)
C6	0.0375 (16)	0.072 (3)	0.068 (2)	0.0045 (17)	0.0072 (15)	0.0029 (19)
C7	0.0363 (16)	0.079 (3)	0.060 (2)	0.0016 (17)	0.0129 (14)	0.0043 (18)
C8	0.0405 (16)	0.0441 (18)	0.0554 (18)	0.0041 (14)	0.0097 (14)	−0.0002 (14)
C9	0.0378 (15)	0.0371 (17)	0.0548 (18)	−0.0002 (13)	0.0093 (13)	0.0008 (14)
C10	0.0448 (16)	0.0393 (17)	0.0534 (18)	−0.0012 (14)	0.0095 (13)	0.0012 (14)
C11	0.0457 (17)	0.057 (2)	0.0503 (18)	−0.0076 (16)	0.0087 (14)	0.0028 (15)
C12	0.0392 (16)	0.059 (2)	0.060 (2)	−0.0053 (16)	0.0118 (14)	−0.0015 (17)
C13	0.0466 (16)	0.0351 (16)	0.0503 (17)	−0.0012 (13)	0.0116 (13)	−0.0015 (13)
C14	0.0369 (15)	0.0408 (17)	0.0514 (18)	0.0015 (13)	0.0093 (13)	−0.0015 (13)
C15	0.0449 (17)	0.063 (2)	0.058 (2)	0.0042 (17)	0.0160 (15)	0.0022 (17)
C16	0.0548 (19)	0.053 (2)	0.052 (2)	−0.0003 (16)	0.0198 (15)	0.0009 (16)
C17	0.0532 (18)	0.0382 (17)	0.0520 (18)	−0.0007 (14)	0.0071 (15)	−0.0029 (14)
C18	0.0388 (15)	0.0405 (17)	0.0544 (19)	−0.0043 (13)	0.0078 (14)	−0.0005 (14)
C19	0.0439 (17)	0.0424 (18)	0.062 (2)	0.0007 (15)	0.0053 (14)	−0.0021 (15)
C20	0.0451 (17)	0.053 (2)	0.061 (2)	−0.0043 (16)	0.0036 (14)	0.0053 (16)
C21	0.057 (2)	0.065 (2)	0.0516 (18)	−0.0027 (18)	0.0019 (15)	0.0040 (17)
C22	0.069 (2)	0.054 (2)	0.055 (2)	−0.0101 (18)	0.0092 (17)	−0.0098 (17)
C23	0.075 (3)	0.063 (3)	0.075 (3)	0.014 (2)	−0.010 (2)	0.003 (2)
C24	0.072 (3)	0.071 (3)	0.081 (3)	−0.019 (2)	−0.011 (2)	0.002 (2)
C25	0.074 (2)	0.0439 (19)	0.065 (2)	−0.0057 (19)	−0.0016 (17)	0.0081 (17)
C26	0.072 (2)	0.057 (2)	0.058 (2)	0.025 (2)	0.0082 (17)	−0.0017 (18)
C27	0.0536 (19)	0.0434 (19)	0.066 (2)	−0.0041 (15)	−0.0010 (16)	0.0010 (16)
C28	0.064 (2)	0.0377 (18)	0.062 (2)	0.0028 (16)	0.0095 (16)	−0.0004 (16)
C29	0.078 (3)	0.066 (3)	0.070 (2)	0.029 (2)	0.002 (2)	−0.006 (2)
C30	0.075 (3)	0.067 (3)	0.075 (2)	0.012 (2)	−0.003 (2)	0.011 (2)

### Geometric parameters (Å, °)

**Table:**

O1—C28	1.351 (4)	C15—C16	1.534 (5)
O1—C13	1.502 (4)	C15—H15A	0.970
O2—C28	1.216 (5)	C15—H15B	0.970
O3—C3	1.421 (4)	C16—C17	1.537 (5)
O3—H3A	0.820	C16—H16A	0.970
C1—C2	1.536 (5)	C16—H16B	0.970
C1—C10	1.547 (4)	C17—C28	1.511 (5)
C1—H1A	0.970	C17—C22	1.520 (5)
C1—H1B	0.970	C17—C18	1.536 (4)
C2—C3	1.506 (5)	C18—C19	1.542 (5)
C2—H2A	0.970	C18—H18A	0.980
C2—H2B	0.970	C19—C29	1.525 (5)
C3—C4	1.537 (5)	C19—C20	1.555 (5)
C3—H3B	0.980	C19—H19A	0.980
C4—C23	1.533 (5)	C20—C30	1.525 (6)
C4—C24	1.537 (5)	C20—C21	1.527 (5)
C4—C5	1.568 (5)	C20—H20A	0.980
C5—C6	1.532 (4)	C21—C22	1.527 (6)
C5—C10	1.556 (4)	C21—H21A	0.970
C5—H5A	0.980	C21—H21B	0.970
C6—C7	1.528 (5)	C22—H22A	0.970
C6—H6A	0.970	C22—H22B	0.970
C6—H6B	0.970	C23—H23A	0.960
C7—C8	1.545 (5)	C23—H23B	0.960
C7—H7A	0.970	C23—H23C	0.960
C7—H7B	0.970	C24—H24A	0.960
C8—C9	1.559 (4)	C24—H24B	0.960
C8—C26	1.562 (5)	C24—H24C	0.960
C8—C14	1.614 (4)	C25—H25A	0.960
C9—C11	1.541 (4)	C25—H25B	0.960
C9—C10	1.569 (4)	C25—H25C	0.960
C9—H9A	0.980	C26—H26A	0.960
C10—C25	1.558 (5)	C26—H26B	0.960
C11—C12	1.522 (5)	C26—H26C	0.960
C11—H11A	0.970	C27—H27A	0.960
C11—H11B	0.970	C27—H27B	0.960
C12—C13	1.510 (4)	C27—H27C	0.960
C12—H12A	0.970	C29—H29A	0.960
C12—H12B	0.970	C29—H29B	0.960
C13—C14	1.563 (4)	C29—H29C	0.960
C13—C18	1.573 (4)	C30—H30A	0.960
C14—C27	1.549 (5)	C30—H30B	0.960
C14—C15	1.552 (4)	C30—H30C	0.960
			
C28—O1—C13	109.7 (2)	C14—C15—H15B	108.6
C3—O3—H3A	109.5	H15A—C15—H15B	107.5
C2—C1—C10	112.7 (3)	C15—C16—C17	113.6 (3)
C2—C1—H1A	109.0	C15—C16—H16A	108.8
C10—C1—H1A	109.0	C17—C16—H16A	108.8
C2—C1—H1B	109.0	C15—C16—H16B	108.8
C10—C1—H1B	109.0	C17—C16—H16B	108.8
H1A—C1—H1B	107.8	H16A—C16—H16B	107.7
C3—C2—C1	112.8 (3)	C28—C17—C22	114.3 (3)
C3—C2—H2A	109.0	C28—C17—C18	98.5 (3)
C1—C2—H2A	109.0	C22—C17—C18	112.6 (3)
C3—C2—H2B	109.0	C28—C17—C16	107.9 (3)
C1—C2—H2B	109.0	C22—C17—C16	113.0 (3)
H2A—C2—H2B	107.8	C18—C17—C16	109.5 (3)
O3—C3—C2	111.3 (3)	C17—C18—C19	113.2 (3)
O3—C3—C4	108.7 (3)	C17—C18—C13	99.6 (2)
C2—C3—C4	113.9 (3)	C19—C18—C13	128.1 (3)
O3—C3—H3B	107.6	C17—C18—H18A	104.6
C2—C3—H3B	107.6	C19—C18—H18A	104.6
C4—C3—H3B	107.6	C13—C18—H18A	104.6
C23—C4—C24	107.6 (3)	C29—C19—C18	112.5 (3)
C23—C4—C3	111.2 (3)	C29—C19—C20	110.6 (3)
C24—C4—C3	107.0 (3)	C18—C19—C20	108.1 (3)
C23—C4—C5	113.5 (3)	C29—C19—H19A	108.5
C24—C4—C5	109.5 (3)	C18—C19—H19A	108.5
C3—C4—C5	107.9 (3)	C20—C19—H19A	108.5
C6—C5—C10	110.2 (3)	C30—C20—C21	109.6 (3)
C6—C5—C4	113.9 (3)	C30—C20—C19	112.0 (3)
C10—C5—C4	118.4 (3)	C21—C20—C19	111.5 (3)
C6—C5—H5A	104.2	C30—C20—H20A	107.9
C10—C5—H5A	104.2	C21—C20—H20A	107.9
C4—C5—H5A	104.2	C19—C20—H20A	107.9
C7—C6—C5	110.7 (3)	C20—C21—C22	113.3 (3)
C7—C6—H6A	109.5	C20—C21—H21A	108.9
C5—C6—H6A	109.5	C22—C21—H21A	108.9
C7—C6—H6B	109.5	C20—C21—H21B	108.9
C5—C6—H6B	109.5	C22—C21—H21B	108.9
H6A—C6—H6B	108.1	H21A—C21—H21B	107.7
C6—C7—C8	114.2 (3)	C17—C22—C21	110.0 (3)
C6—C7—H7A	108.7	C17—C22—H22A	109.7
C8—C7—H7A	108.7	C21—C22—H22A	109.7
C6—C7—H7B	108.7	C17—C22—H22B	109.7
C8—C7—H7B	108.7	C21—C22—H22B	109.7
H7A—C7—H7B	107.6	H22A—C22—H22B	108.2
C7—C8—C9	108.9 (3)	C4—C23—H23A	109.5
C7—C8—C26	106.2 (3)	C4—C23—H23B	109.5
C9—C8—C26	110.9 (3)	H23A—C23—H23B	109.5
C7—C8—C14	109.7 (3)	C4—C23—H23C	109.5
C9—C8—C14	108.8 (2)	H23A—C23—H23C	109.5
C26—C8—C14	112.2 (3)	H23B—C23—H23C	109.5
C11—C9—C8	109.3 (3)	C4—C24—H24A	109.5
C11—C9—C10	114.6 (3)	C4—C24—H24B	109.5
C8—C9—C10	117.0 (2)	H24A—C24—H24B	109.5
C11—C9—H9A	104.9	C4—C24—H24C	109.5
C8—C9—H9A	104.9	H24A—C24—H24C	109.5
C10—C9—H9A	104.9	H24B—C24—H24C	109.5
C1—C10—C5	106.7 (3)	C10—C25—H25A	109.5
C1—C10—C25	107.4 (3)	C10—C25—H25B	109.5
C5—C10—C25	114.3 (3)	H25A—C25—H25B	109.5
C1—C10—C9	108.2 (3)	C10—C25—H25C	109.5
C5—C10—C9	107.1 (2)	H25A—C25—H25C	109.5
C25—C10—C9	112.7 (3)	H25B—C25—H25C	109.5
C12—C11—C9	111.4 (3)	C8—C26—H26A	109.5
C12—C11—H11A	109.3	C8—C26—H26B	109.5
C9—C11—H11A	109.3	H26A—C26—H26B	109.5
C12—C11—H11B	109.3	C8—C26—H26C	109.5
C9—C11—H11B	109.3	H26A—C26—H26C	109.5
H11A—C11—H11B	108.0	H26B—C26—H26C	109.5
C13—C12—C11	114.7 (3)	C14—C27—H27A	109.5
C13—C12—H12A	108.6	C14—C27—H27B	109.5
C11—C12—H12A	108.6	H27A—C27—H27B	109.5
C13—C12—H12B	108.6	C14—C27—H27C	109.5
C11—C12—H12B	108.6	H27A—C27—H27C	109.5
H12A—C12—H12B	107.6	H27B—C27—H27C	109.5
O1—C13—C12	104.9 (3)	O2—C28—O1	120.0 (3)
O1—C13—C14	106.9 (2)	O2—C28—C17	130.0 (3)
C12—C13—C14	113.2 (3)	O1—C28—C17	110.0 (3)
O1—C13—C18	97.8 (2)	C19—C29—H29A	109.5
C12—C13—C18	115.0 (3)	C19—C29—H29B	109.5
C14—C13—C18	116.7 (2)	H29A—C29—H29B	109.5
C27—C14—C15	107.5 (3)	C19—C29—H29C	109.5
C27—C14—C13	107.8 (3)	H29A—C29—H29C	109.5
C15—C14—C13	108.3 (3)	H29B—C29—H29C	109.5
C27—C14—C8	111.0 (3)	C20—C30—H30A	109.5
C15—C14—C8	110.5 (3)	C20—C30—H30B	109.5
C13—C14—C8	111.5 (2)	H30A—C30—H30B	109.5
C16—C15—C14	114.9 (3)	C20—C30—H30C	109.5
C16—C15—H15A	108.6	H30A—C30—H30C	109.5
C14—C15—H15A	108.6	H30B—C30—H30C	109.5
C16—C15—H15B	108.6		

### Hydrogen-bond geometry (Å, °)

**Table:**

*D*—H···*A*	*D*—H	H···*A*	*D*···*A*	*D*—H···*A*
O3—H3A···O2^i^	0.82	2.24	3.059 (4)	176

Symmetry codes: (i) −*x*, *y*+1/2, −*z*+2.
